# Inflammasomes and Signaling Pathways: Key Mechanisms in the Pathophysiology of Sepsis

**DOI:** 10.3390/cells14120930

**Published:** 2025-06-19

**Authors:** Jhan S. Saavedra-Torres, María Virginia Pinzón-Fernández, Martin Ocampo-Posada, H. A. Nati-Castillo, Laura Alejandra Jiménez Hincapie, Eder J. Cadrazo-Gil, Marlon Arias-Intriago, Marlon Rojas-Cadena, Andrea Tello-De-la-Torre, Walter Osejos, Juan S. Izquierdo-Condoy

**Affiliations:** 1 Grupo de Investigación en Salud (GIS), Universidad del Cauca, Popayan 190002, Colombia; 2 Grupo de Investigación en Ciencias Básicas y Clínicas de la Salud, Universidad Javeriana, Cali 760031, Colombia; 3 Interinstitutional Group of Internal Medicine (GIMI I), Universidad Libre, Cali 760031, Colombia; 4 Facultad de Medicina, Fundación Universitaria Autónoma de las Américas, Pereira 660003, Colombia; 5 Facultad de Medicina, Universidad del Norte, Barranquilla 081007, Colombia; 6 One Health Research Group, Universidad de las Américas, Quito 170124, Ecuador; 7 Departamento de Medicina Interna, Clínica Guayaquil, Guayaquil 090313, Ecuador

**Keywords:** sepsis, inflammasome, pyroptosis, cytokines, coagulation, immunomodulation

## Abstract

Sepsis is a life-threatening syndrome characterized by a dysregulated immune response to infection, frequently leading to multiorgan failure and high mortality. Inflammasomes—cytosolic multiprotein complexes of the innate immune system—serve as critical platforms for sensing pathogen- and damage-associated molecular patterns (PAMPs and DAMPs). Key sensors such as NLRP3, AIM2, and IFI16 initiate caspase-1 activation, IL-1β and IL-18 maturation, and gasdermin D–mediated pyroptosis. In sepsis, excessive inflammasome activation drives oxidative stress, endothelial dysfunction, immunothrombosis, and immune exhaustion. This maladaptive cascade is further aggravated by the release of DAMPs and procoagulant factors, compromising vascular integrity and immune homeostasis. Prolonged activation contributes to immunoparalysis, lymphopenia, and increased susceptibility to secondary infections. Inflammasome signaling also intersects with necroptosis and ferroptosis, amplifying systemic inflammation and tissue injury. Additionally, various pathogens exploit immune evasion strategies to modulate inflammasome responses and enhance virulence. Therapeutic interventions under investigation include selective NLRP3 inhibitors, IL-1 blockers, gasdermin D antagonists, and extracorporeal cytokine hemoadsorption. Emerging approaches emphasize biomarker-guided immunomodulation to achieve personalized therapy. While preclinical studies have shown promising results, clinical translation remains limited. Targeting inflammasomes may offer a path toward precision immunotherapy in sepsis, with potential to reduce organ dysfunction and improve survival.

## 1. Introduction

The inflammasome is a cytosolic multiprotein complex that plays a pivotal role in the innate immune response by functioning as a molecular sensor of pathogen-associated molecular patterns (PAMPs) and damage-associated molecular patterns (DAMPs). Upon activation, it initiates the maturation and secretion of proinflammatory cytokines—interleukin-1 beta (IL-1β) and interleukin-18 (IL-18)—and triggers pyroptosis, a highly inflammatory form of programmed cell death [1,2,3]. Among the various inflammasome complexes identified, the NLRP3 inflammasome (NOD-like receptor family pyrin domain-containing 3) is the most extensively studied and clinically significant in the context of sepsis.

Sepsis is a life-threatening condition resulting from a dysregulated host response to infection, affecting approximately 49 million individuals globally and accounting for over 11 million deaths annually [4,5]. The syndrome is characterized by a complex interplay of hyperinflammation, immune exhaustion, endothelial dysfunction, and coagulopathy. Recent transcriptomic analyses of septic patients have revealed sustained activation of inflammasome-associated components, including caspase-1 and gasdermin D (GSDMD), in circulating monocytes. This activation correlates with organ dysfunction and adverse outcomes [5,6,7]. Additionally, elevated plasma levels of IL-1β and IL-18 have been independently associated with increased mortality [5]. Experimental models deficient in NLRP3 or the adaptor protein ASC (apoptosis-associated speck-like protein containing a CARD) exhibit reduced cytokine release, attenuated organ injury, and markedly improved survival, underscoring the therapeutic potential of targeting inflammasome pathways [2,8,9,10,11].

Notably, NLRP3 activation also contributes to the immunosuppressive phase of sepsis by promoting immune cell exhaustion and metabolic reprogramming, which perpetuate long-term immune dysfunction [12,13]. Thus, the inflammasome serves not only as a mediator of acute inflammation but also as a central driver of chronic immune derangement.

This review aims to integrate current knowledge on the molecular mechanisms, pathophysiological consequences, and therapeutic implications of inflammasome activation in sepsis.

## 2. Molecular Basis of Inflammasome Activation in Sepsis

### 2.1. Toll-like Receptors as Upstream Sensors in Sepsis

Toll-like receptors (TLRs) are pivotal pattern recognition receptors (PRRs) of the innate immune system that serve as primary sensors for microbial components and endogenous danger signals during sepsis. Expressed predominantly on monocytes, macrophages, dendritic cells, and epithelial cells, TLRs initiate the host’s first line of defense against infection and tissue damage [14,15,16]. Although viral infections activate specialized cytosolic pathways such as RIG-I/MAVS and cGAS-STING, TLRs also contribute significantly to antiviral responses through the recognition of nucleic acids by endosomal receptors like TLR3, TLR7, and TLR8 [17,18].

In bacterial sepsis, TLR4 and TLR2 play central roles by recognizing lipopolysaccharide (LPS) and bacterial lipoproteins, respectively, thereby initiating a robust inflammatory response through adaptor proteins such as MyD88 and TRIF. This signaling cascade promotes the release of type I interferons, proinflammatory cytokines, and the upregulation of co-stimulatory molecules necessary for effective immune activation [19]. Beyond their role in infection sensing, TLRs are also activated by DAMPs released during sterile tissue injury, amplifying the inflammatory response even in the absence of pathogens.

This dual ability to detect both PAMPs and DAMPs positions TLRs as key immunological hubs in sepsis pathophysiology. Their overactivation contributes to systemic inflammation, vascular dysfunction, and progression to multiple organ failure. Therefore, TLRs represent critical upstream mediators in sepsis, whose dysregulated signaling sets the stage for downstream inflammasome activation and metabolic derangement [20,21,22].

### 2.2. Key Sensor of Metabolic and Immune Balance

The NLRP3 inflammasome plays a physiological role beyond microbial defense. Its controlled activation enables the regulated release of IL-1β and IL-18, which are essential for rapid immune responses and tissue repair. In mucosal sites such as the intestine, NLRP3 acts as both a metabolic and immunologic sensor that sustains microbiota balance, restricts pathogenic colonization, and preserves epithelial integrity. At the systemic level, it contributes to the clearance of damaged cells, adaptation to mitochondrial stress, and regulation of inflammaging [23,24].

In sepsis, however, inflammasome regulation becomes impaired. Excessive activation driven by PAMPs and DAMPs intensifies systemic inflammation, induces pyroptosis in immune and endothelial cells, disrupts epithelial barriers, and promotes multiorgan dysfunction. Metabolic alterations and intestinal dysbiosis further enhance this dysregulated activation [25]. Thus, while protective under homeostatic conditions, inflammasome overactivation in sepsis becomes a critical mediator of irreversible tissue damage [24,26].

### 2.3. Social Dimensions of Inflammation

The pathophysiology of sepsis is shaped by biological and social determinants. Inflammasome activation integrates infectious, metabolic, and environmental signals, but is influenced by host context [27,28]. In low-socioeconomic populations, chronic exposure to microinflammation, obesity, and environmental pollutants may lead to a pre-activated inflammatory state, predisposing individuals to exaggerated responses during severe infections [29,30,31].

In neonates, limited inflammasome activity results from immunological immaturity, characterized by poor memory responses, reduced complement activation, and a predominantly anti-inflammatory profile [32,33]. While this increases susceptibility to infection, it paradoxically provides initial protection against hyperinflammation [34]. In contrast, older adults experience immunosenescence, marked by reduced pathogen control and persistent baseline inflammation (inflammaging), which sensitizes inflammasomes to overactivation [35,36]. Additionally, comorbidities such as hypertension and diabetes—prevalent in impoverished settings—promote endothelial dysfunction, creating a microenvironment prone to vascular barrier breakdown during sepsis [28,37,38]. These factors position the inflammasome as both a molecular sensor and a sociobiological integrator of inflammatory risk.

#### Sex-Based Differences in Inflammasome Regulation

The NLRP3 inflammasome also exhibits sex-dependent regulation. Estrogen, especially 17β-estradiol, modulates NLRP3 activity via G protein-coupled estrogen receptor 1 (GPER-1) [39,40]. Upon activation, GPER-1 promotes nuclear translocation of peroxisome proliferator-activated receptor delta (PPARδ), which suppresses NLRP3 activation. This pathway has been proposed to contribute to lower sepsis mortality in women [41].

In a prospective ICU study, sepsis survivors showed significantly higher GPER-1 concentrations and more favorable clinical parameters compared to nonsurvivors, including higher platelet counts, CRP levels, SOFA, and APACHE II scores—all positively correlated with GPER-1 levels [41,42].

This mechanism is particularly relevant in premenopausal women, in whom estradiol enhances the anti-inflammatory effect via GPER-1. The result is reduced NLRP3 activation, oxidative stress mitigation, and improved cardiovascular function. While not the sole explanation, the GPER-1/PPARδ axis offers a compelling rationale for sex differences in sepsis outcomes, including reduced mortality, vasopressor requirements, and faster hemodynamic recovery [39,40,42].

### 2.4. Canonical and Non-Canonical Pathways

A central outcome of this activation is the proteolytic processing of the inactive precursors of IL-1β and IL-18 into their mature, bioactive forms by caspase-1 [43,44,45].

The canonical inflammasome pathway involves a sensor protein—such as NLRP1—along with the adaptor ASC and pro-caspase-1. Upon stimulation by triggers including LPS, cytosolic DNA, or various forms of cellular stress, the sensor undergoes oligomerization and recruits ASC, promoting proximity-induced activation of caspase-1 [43,44,45].

Non-canonical pathways also play a substantial role in inflammasome activation. In humans, caspase-5 (homologous to murine caspase-11) is directly activated by intracellular LPS. Once activated, these caspases cleave GSDMD, leading to pore formation in the cell membrane and pyroptotic cell death, thereby amplifying inflammation independently of canonical inflammasome sensors [46,47].

Consequently, the inflammasome is not only a key component of host defense but also a central mediator of sepsis pathogenesis. Dysregulation of both canonical and non-canonical pathways contributes to excessive cytokine production, pyroptosis, and multiorgan failure. Moreover, the inflammasome interacts with other regulated cell death mechanisms such as necroptosis and ferroptosis, creating a self-perpetuating inflammatory loop that exacerbates systemic injury in sepsis [44,45].

### 2.5. Pyroptosis and Inflammatory Amplification

Caspase-1 activation leads to cleavage of GSDMD, whose N-terminal domain forms membrane pores [43,44,45], enabling the release of IL-1β and IL-18 and triggering pyroptosis—a lytic, proinflammatory form of programmed cell death. This process contributes to the rapid escalation of inflammation and the release of DAMPs, which further recruit and activate immune cells [46,47].

Experimental models using THP-1 monocytic cells have shown that membrane disruption alone is sufficient to activate inflammasome components. Immunodepletion of ASC completely abrogates this response, highlighting its essential scaffolding function. Biochemical studies confirm that upon inflammatory stimulation, NALP1, ASC, and caspase-1 assemble into high-molecular-weight complexes [45,48].

**Figure 1:**
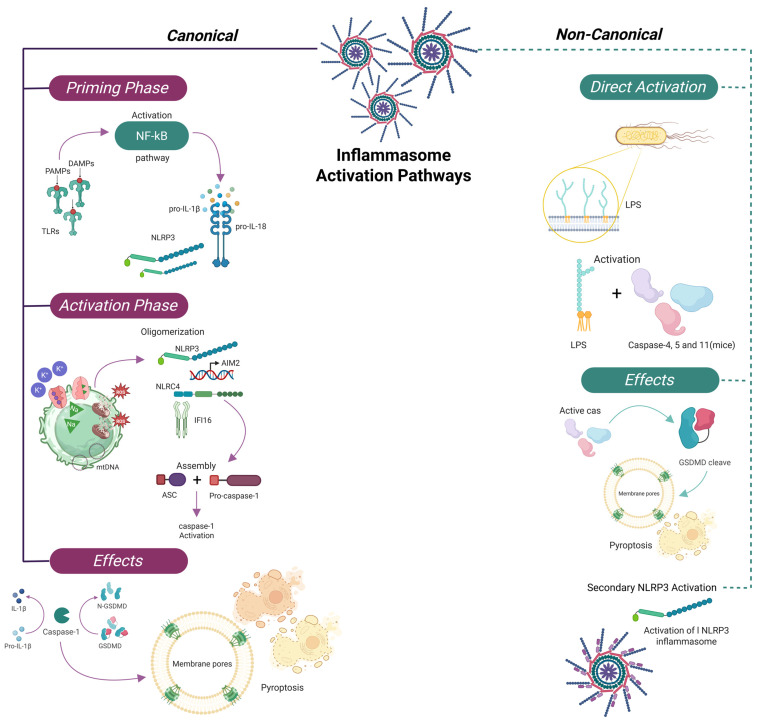
Inflammasome activation pathways—canonical and non-canonical. This diagram illustrates the two major pathways of inflammasome activation: the canonical route, driven by pattern recognition receptors such as NLRP3, AIM2, NLRC4, and IFI16, and the non-canonical route involving direct sensing of intracellular LPS by caspases-4/5 in humans (caspase-11 in mice). The canonical pathway requires a priming phase via NF-κB activation, leading to pro-IL-1β and pro-IL-18 expression, followed by an activation phase that triggers inflammasome assembly and caspase-1 activation. The non-canonical pathway bypasses this priming step and directly activates inflammatory caspases, leading to GSDMD cleavage, pore formation, pyroptosis, and secondary NLRP3 activation. This integrated response links microbial detection with cytokine release and regulated cell death, playing a central role in immune defense and sepsis pathogenesis.

While inflammasome activation serves as a critical host defense mechanism, its dysregulation in sepsis leads to excessive cytokine release, widespread pyroptosis, and progressive multiorgan dysfunction. In this context, the inflammasome acts not only as a sentinel of cellular stress but also as a molecular amplifier of systemic inflammatory damage [45,48]. In addition to pyroptosis, inflammasomes interact with other regulated cell death pathways, such as necroptosis and ferroptosis, which amplify tissue damage and systemic inflammation in sepsis. These interactions represent an emerging area of research with therapeutic implications [49,50] (Figure 1).

### 2.6. Necroptosis, Pyroptosis, and Ferroptosis in Sepsis

Sepsis involves concurrent activation of necroptosis, pyroptosis, and ferroptosis, all contributing to multiorgan failure. Elevated RIPK3, a necroptosis marker, correlates with vasopressor dependence and mechanical ventilation in septic shock [51,52]. Pyroptosis, via NLRP3 activation and GSDMD cleavage, releases IL-1β and IL-18, with serum levels correlating with SOFA scores and mortality [49,52,53].

Regarding ferroptosis, recent studies show an accumulation of free iron and a decrease in GPX4 in critical tissues such as the lung and kidney during sepsis. This promotes lipid peroxidation and organ failure. These processes generate a sustained inflammatory storm, aggravate the cellular redox imbalance, and hinder tissue repair [54]. Pharmacological blockade of these pathways—particularly with RIPK1 inhibitors, lipophilic antioxidants, or inflammasome antagonists—represents a promising strategy to attenuate sepsis progression and improve clinical outcomes [55,56].

### 2.7. Pyroptosis and Non-Classic Inflammasomes

Pyroptosis is a form of explosive, lytic cell death that functions as a defense mechanism against intracellular infections. Unlike apoptosis, pyroptosis is abrupt and inflammatory, characterized by cytokine release and membrane rupture. It is initiated by inflammasome activation, and its execution depends on effector proteins such as gasdermins [57,58].

Among these, GSDMD is the most well-studied. However, in oncological or non-infectious contexts, other family members such as GSDME and GSDMB may also be involved. These are activated by apoptotic caspases or granzymes, broadening the functional scope of pyroptosis beyond classical innate immunity [57,59].

Recent optogenetic studies have revealed that pyroptosis unfolds in two sequential phases: initial ionic shifts followed by NINJ1-mediated plasma membrane rupture. The intensity of the inflammasomal stimulus influences both the kinetics of cell death and the profile of DAMPs released—an especially relevant factor in sepsis, where excessive activation leads to widespread tissue injury. Thus, inflammasome-driven pyroptosis acts not only as a form of cell death but also as a potent immune alarm system [57,60].

Additionally, non-classical inflammasomes can induce pyroptosis through caspase-4/5/11 activation. This pathway facilitates the clearance of infected or damaged cells while amplifying local inflammation, linking inflammasome signaling to systemic manifestations of sepsis, including fever, vasodilation, and immune-mediated tissue damage [57,59,60].

## 3. Key Inflammasome Sensors in Sepsis

Distinct inflammasome sensors detect specific classes of danger signals and pathogens. The following sections describe key sensors implicated in sepsis.

### 3.1. NLRP3: Central Integrator of Danger Signals

The NLRP3 inflammasome serves as a critical signaling hub in sepsis by integrating diverse endogenous and exogenous stress signals. It is activated by a broad range of stimuli, including metabolic stress (e.g., hypoxia), mitochondrial dysfunction leading to mitochondrial DNA (mtDNA) and reactive oxygen species (ROS) release, and ionic disturbances such as potassium efflux (K^+^) and calcium influx (Ca^2+^). Additionally, lysosomal destabilization caused by crystals or microbial toxins results in the release of cathepsins, which further amplify NLRP3 activation [61,62,63].

This convergence of signals enables NLRP3 to amplify inflammatory cascades, promote tissue injury, and drive progression toward multiorgan dysfunction. Beyond inflammation, NLRP3 contributes to sepsis-associated coagulopathy by inducing tissue factor expression and phosphatidylserine externalization—mechanisms that initiate coagulation and microthrombus formation [6,64,65].

In contrast, NLRP1b is activated by microbial proteases, particularly the lethal toxin of *Bacillus anthracis*, which cleaves its N-terminal domain. This cleavage marks the protein for proteasomal degradation via the N-end rule pathway, liberating the C-terminal fragment to assemble the inflammasome and activate caspase-1, leading to IL-1β and IL-18 maturation and pyroptosis [66,67,68].

Alternative activation mechanisms involve caspase-8, which, in response to TLR4 or TNF receptor signaling, can cleave NLRP1b, reinforcing inflammatory responses through an amplification loop [69,70,71].

In parallel, Pyrin serves as another important sensor activated by bacterial toxins—such as *Clostridium difficile* toxin B—that inactivate Rho GTPases, thereby disrupting cytoskeletal homeostasis. Rather than recognizing microbial ligands directly, Pyrin senses pathogen-induced alterations in host cell function. This leads to the recruitment of ASC, activation of caspase-1, and release of IL-1β and IL-18. Both NLRP1b and Pyrin play crucial roles in the innate immune recognition of intracellular pathogens during sepsis [71,72,73,74] (Table 1).

#### 3.1.1. Post-Translational Regulation of NLRP3 Activation

The activation of NLRP3 is tightly regulated by multiple post-translational modifications (PTMs), which function as molecular switches to modulate its activity in response to environmental and intracellular cues. Phosphorylation at conserved residues within the pyrin domain alters the electrostatic surface of NLRP3, facilitating the oligomerization necessary for inflammasome assembly [75,76,77]. This is counterbalanced by phosphatases, which remove phosphate groups to prevent inappropriate activation.

Ubiquitination exerts both inhibitory and permissive effects. K48- and K63-linked polyubiquitin chains typically target NLRP3 for degradation via the proteasome or autophagy pathways. Specific E3 ubiquitin ligases can inhibit inflammasome assembly by destabilizing NLRP3, whereas deubiquitinases (DUBs) stabilize the cytoplasmic pool of NLRP3 by removing ubiquitin chains, preserving its readiness for activation [75,78].

SUMOylation, particularly within the NACHT domain, acts as a negative regulator that maintains NLRP3 in an inactive conformation under basal conditions. This reversible modification prevents premature inflammasome assembly, serving as a fail-safe mechanism against aberrant inflammation [75,76].

A recently described PTM, UFMylation, involves the conjugation of ubiquitin-fold modifier 1 (UFM1) to NLRP3 during the priming phase. This modification interferes with K63-linked ubiquitination, thereby protecting NLRP3 from autophagic degradation. Loss of UFMylation in myeloid cells has been shown to impair inflammasome assembly, reduce cytokine secretion, and attenuate tissue injury in experimental models—underscoring its importance in inflammasome regulation [75,76].

Together, these PTMs determine the activation threshold, subcellular localization, and stability of NLRP3. By dynamically integrating signals of cellular stress and infection, these regulatory mechanisms ensure that inflammasome activation is both precise and context-dependent [76,77].

#### 3.1.2. Ionic Dynamics of the Inflammasome

Potassium (K^+^) efflux is a critical intracellular signal in NLRP3 inflammasome activation, common to numerous inflammatory stimuli [61,79,80]. Sustained cytosolic K^+^ depletion induces a conformational change in NLRP3 that promotes its interaction with ASC and procaspase-1, enabling formation of the active inflammasome complex [61,79]. This precedes gasdermin D cleavage and the release of proinflammatory cytokines such as IL-1β and IL-18, marking the onset of pyroptosis.

Several ion channels and membrane proteins—such as P2X7, GSDMD, pannexin-1, and K2P channels—are involved in regulating these fluxes. Their modulation has therapeutic potential in inflammatory diseases. Although calcium (Ca^2+^) influx is not universally required for NLRP3 activation, it acts as a potentiating cofactor in non-immune cells by enhancing inflammatory signaling [61,80]. Sodium (Na^+^) influx contributes to osmotic imbalance but is not essential for inflammasome assembly.

Understanding these ionic dynamics allows the integration of immunological mechanisms with biophysical parameters, opening new avenues for pharmacologic intervention in uncontrolled systemic inflammation, such as that seen in severe sepsis [61,79,80].

### 3.2. AIM2: Cytosolic DNA Sensor

Absent in Melanoma 2 (AIM2) is a cytosolic pattern recognition receptor that detects double-stranded DNA (dsDNA) via its HIN200 domain. Upon sensing cytosolic DNA, AIM2 engages the adaptor protein ASC through its pyrin domain, forming an inflammasome complex that activates caspase-1 [81,82]. This activation leads to the cleavage of pro–IL-1β and pro–IL-18 into their mature forms, and the processing of GSDMD, whose N-terminal fragment forms membrane pores that initiate pyroptosis. This inflammatory form of programmed cell death facilitates the release of proinflammatory cytokines and DAMPs, thereby amplifying innate immune responses [81,82].

AIM2 is essential for host defense against intracellular bacteria such as *Francisella* and *Brucella*, as well as DNA viruses including vaccinia and SARS-CoV-2. However, in the context of sepsis—characterized by dysregulated systemic inflammation—persistent microbial or mtDNA may aberrantly activate AIM2, leading to uncontrolled cytokine production and immune exhaustion. Experimental models demonstrate that silencing AIM2 or its downstream effectors significantly reduces IL-1β levels and mitigates tissue damage, highlighting its potential as a therapeutic target in sepsis [81,82].

AIM2 activation is further potentiated by factors commonly observed in septic infections, such as mitochondrial ROS and endosomal membrane disruption. This is particularly relevant in infections caused by *Francisella*, *Brucella*, Influenza virus, and SARS-CoV, which promote these intracellular disturbances and thus facilitate inflammasome assembly [10,83,84,85].

### 3.3. IFI16: Nuclear DNA Sensor

Interferon gamma-inducible protein 16 (IFI16) is a nuclear PYHIN sensor that detects non-integrated viral DNA within the nucleus of infected cells. Unlike cytosolic sensors such as NLRP3 and AIM2, IFI16 is unique in its nuclear localization and its ability to sense nuclear viral genomes. Upon DNA binding through its HIN200 domain, IFI16 recruits ASC via its pyrin domain, forming a nuclear inflammasome that activates caspase-1 and triggers downstream inflammatory signaling [86,87].

This mechanism is well-characterized in latent Kaposi’s sarcoma-associated herpesvirus (KSHV) infection, where IFI16 colocalizes with the viral genome and promotes IL-1β release. Interestingly, IFI16 has been shown to translocate from the nucleus to the cytoplasm under certain conditions, suggesting a dynamic, spatial regulation of inflammasome activity. Gene silencing of IFI16 or ASC impairs caspase-1 activation and cytokine maturation, reinforcing their essential role in viral sensing and inflammatory signaling [86,87].

Importantly, IFI16-mediated responses are specific to nuclear-replicating DNA viruses such as KSHV and not triggered by cytoplasmic viruses like vaccinia. This selectivity may underlie IFI16’s contribution to chronic immunopathology in persistent viral infections and their role in sepsis-related tissue damage [86,87].

### 3.4. NLRC4: Pathogen-Induced Pyroptosis

The NLRC4 inflammasome, also known as IPAF, is a cytosolic sensor that detects intracellular bacterial components, especially those delivered by the type III secretion systems (T3SS) of pathogens such as *Salmonella enterica* serovar Typhimurium. One key effector, SipB, facilitates NLRC4 oligomerization and recruitment of ASC and pro–caspase-1, initiating inflammasome assembly [48,88].

Activated caspase-1 then processes pro–IL-1β and pro–IL-18 and cleaves GSDMD, whose N-terminal fragment forms pores (1.1–2.4 nm) in the plasma membrane. These pores disrupt osmotic balance, causing water influx, cytosolic content release, and cell swelling, culminating in pyroptosis [48,81,89]. Unlike apoptosis, pyroptosis is marked by caspase-1-dependent DNA fragmentation, independent of the classical ICAD/CAD pathway, likely involving an unidentified nuclease [48,88].

Notably, IL-1β and IL-18 may be secreted through GSDMD pores before complete membrane rupture, allowing early cytokine release without immediate cell lysis. Once pyroptosis is complete, additional DAMPs such as HMGB1 and LDH are released, further amplifying systemic inflammation [48,81,89].

Although NLRC4 was initially characterized in response to *Salmonella*, it also responds to other pathogens that utilize similar secretion systems. In sepsis, sustained or dysregulated activation of the NLRC4–caspase-1 axis can contribute to excessive pyroptosis, systemic inflammation, and multiorgan failure [48,81,89] (Table 2).

## 4. Pathophysiological Consequences of Inflammasome Activation

### 4.1. CGAS-STING Pathway in Sepsis

The cyclic GMP–AMP synthase–stimulator of interferon genes (cGAS–STING) pathway plays a pivotal role in the inflammatory pathophysiology of sepsis, functioning as a molecular bridge between mitochondrial damage, cytosolic DNA detection, and the amplification of innate immune responses. Under conditions of cellular stress, opening of the mitochondrial permeability transition pore (mPTP) permits the release of mitochondrial DNA (mtDNA) into the cytosol [84,85]. This mtDNA serves as a DAMP, which activates cGAS to produce cyclic GMP–AMP (cGAMP), thereby triggering STING activation. Once activated, STING translocates to the Golgi apparatus and initiates a signaling cascade through phosphorylation of TANK-binding kinase 1 (TBK1) and interferon regulatory factor 3 (IRF3), ultimately leading to the transcription of type I interferons [90,91,92].

Beyond its canonical antiviral function, the cGAS–STING pathway potentiates inflammasome activation, particularly of NLRP3, thereby enhancing caspase-1 activation, GSDMD cleavage, pyroptosis, and the secretion of IL-1β and IL-18. In sepsis, persistent mitochondrial dysfunction drives chronic activation of this pathway, promoting widespread immune cell death and uncontrolled inflammation. The cGAS–STING–NLRP3 axis thereby amplifies both sterile and pathogen-induced inflammation, contributing to progressive tissue damage and multiorgan dysfunction. Notably, inflammasome components such as apoptosis-associated speck-like protein containing a CARD (ASC) and potassium flux-regulating ion channels further modulate this pathway, establishing a self-reinforcing proinflammatory loop that sustains the immunopathology of sepsis [90,93].

### 4.2. Gasdermin D and Inflammatory Transcription

GSDMD activation represents a key intersection between inflammatory cell death and gene regulation during immune activation. Upon infectious or inflammatory stimuli, caspase-1 (canonical pathway) or caspases-4/5/11 (non-canonical pathway) cleave GSDMD, releasing its N-terminal fragment. This fragment inserts into the plasma membrane, forming pores that permit the release of IL-1β, IL-18, and DAMPs, generating a proinflammatory microenvironment [94,95].

These cytokines activate signaling cascades in neighboring cells, including NF-κB, JAK–STAT, and IRF pathways. IRF2, in particular, promotes GSDMD gene transcription, establishing a feedback loop that amplifies the inflammatory response [85,87]. Thus, GSDMD not only disrupts membrane integrity but also influences transcriptional programs in damaged tissues [95,96].

### 4.3. Oxidative Stress and Endothelial Dysfunction

During sepsis, inflammasome activation is closely linked to oxidative stress and mitochondrial dysfunction induced by systemic infection [92,97,98]. Damaged mitochondria release ROS and mtDNA, both of which act as DAMPs. ROS oxidize the thioredoxin (TRX) protein, resulting in the release of thioredoxin-interacting protein (TXNIP), which then binds to and activates the NLRP3 inflammasome [92,99,100]. Concurrently, cytosolic mtDNA can activate the AIM2 inflammasome or engage the cyclic GMP–AMP synthase–stimulator of interferon genes (cGAS–STING) pathway, promoting type I interferon responses that, although initially protective, can become pathogenic if sustained [101,102]. These converging signals drive caspase-1 activation, maturation of IL-1β and IL-18, and pyroptosis. The resulting proinflammatory environment contributes to endothelial dysfunction, activation of the coagulation cascade, and immunoparalysis—key events in the progression of sepsis [103,104].

Under septic conditions, antioxidant defenses—such as glutathione, superoxide dismutase, and catalase—are rapidly depleted. Excess ROS damage lipids, proteins, and nucleic acids, leading to lipid peroxidation and the generation of cytotoxic aldehydes like 4-hydroxynonenal (4-HNE) [62,105,106]. These by-products compromise membrane integrity, increase permeability, and contribute to both apoptotic and necrotic cell death. In parallel, ROS activate transcription factors including NF-κB and AP-1, which further amplify inflammation by inducing TNF-α and IL-6 expression [107,108,109].

From a vascular perspective, inflammasome activation contributes directly to endothelial dysfunction, a pivotal event in septic shock. Endothelial cells upregulate adhesion molecules (e.g., ICAM-1, VCAM-1), facilitating leukocyte adhesion, transmigration, and microvascular thrombosis. The combined effects of ROS, IL-1β, and cytokines increase capillary permeability, hypoperfusion, and hypotension—defining features of advanced sepsis [107,110,111,112].

### 4.4. Inflammation–Coagulation Axis and Immunothrombosis

The inflammasome plays a central role in the intersection between inflammation and coagulation, a process known as immunothrombosis. Caspase-1–dependent IL-1β release and GSDMD cleavage promote the externalization of phosphatidylserine and the expression of tissue factor (TF) on monocytes and epithelial cells. This initiates the extrinsic coagulation cascade, leading to thrombin generation, platelet activation, fibrin deposition, and ultimately microvascular thrombosis and disseminated intravascular coagulation (DIC)—a life-threatening complication of sepsis [113,114,115].

A similar prothrombotic profile is observed in atherosclerosis, where chronic inflammation and immune dysregulation promote coagulation. The immune-responsive gene 1 (IRG1)–itaconate axis plays a regulatory role: IRG1 is upregulated in atherosclerotic plaques, and its deficiency increases lipid accumulation, IL-1β production, NET formation, and NLRP3 activation. Conversely, treatment with 4-octyl itaconate (4-OI) reduces systemic inflammation and improves vascular pathology in experimental models [116,117].

Simultaneously, the renin–angiotensin–aldosterone system (RAAS) exacerbates endothelial dysfunction through angiotensin II–mediated oxidative stress, cytokine release, and TF expression, promoting a prothrombotic state. This mechanism is particularly relevant in infections like COVID-19 [118]. Inflammation-induced thrombosis is perpetuated by a bidirectional feedback loop in which thrombin and activated platelets further enhance cytokine production and vascular inflammation. Integrated therapeutic strategies—such as combining immunometabolic regulators (e.g., 4-OI) with RAAS inhibitors and antithrombotic agents—may provide synergistic control over inflammation, thrombosis, and immune dysregulation in sepsis and related disorders [116,117,118].

### 4.5. Immunoparalysis and Secondary Infections

While inflammasome activation is crucial for initiating antimicrobial responses, its persistent stimulation leads to immunoparalysis—a maladaptive immunosuppressive state. This phase is marked by lymphopenia, apoptosis of effector T cells, reduced HLA-DR expression on monocytes, and upregulation of immune checkpoint molecules such as PD-1 and CTLA-4. These alterations impair antigen presentation, promote T-cell exhaustion, and compromise pathogen clearance. Monocytes and macrophages undergo functional reprogramming toward a tolerogenic phenotype, characterized by diminished TNF-α and IL-12 production and reduced microbial killing. This phenotype is sustained by a metabolic shift toward oxidative phosphorylation, reduced glycolysis, and epigenetic silencing of proinflammatory gene expression. Natural killer (NK) cells also show reduced cytotoxicity and IFN-γ production, while dendritic cells exhibit impaired antigen presentation and fail to activate naïve T cells, further weakening adaptive immune responses [88,119,120,121].

**Figure 2:**
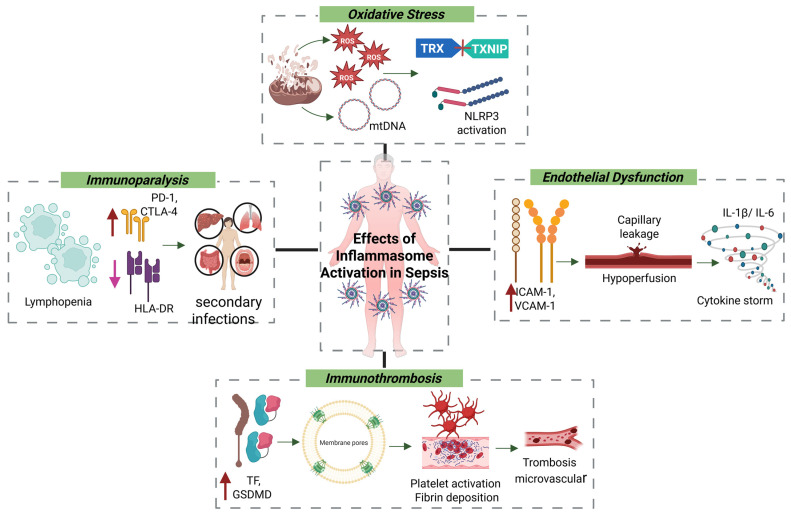
Pathophysiological Consequences of Inflammasome Activation in Sepsis. This schematic illustrates the major downstream effects of inflammasome activation in sepsis, including oxidative stress (mediated by mitochondrial damage and ROS release), endothelial dysfunction (increased permeability and cytokine storm), immunothrombosis (gasdermin D-mediated pore formation and coagulation), and immunoparalysis (lymphopenia and immune checkpoint upregulation). Together, these processes drive tissue injury, multiorgan dysfunction, and immune exhaustion characteristic of advanced septic states.

Clinically, this immunosuppression increases susceptibility to secondary infections, delays wound healing, and promotes reactivation of latent viruses such as CMV and HSV. It is also associated with increased late-stage mortality in sepsis. Contributing factors include prolonged hospitalization, mechanical ventilation, and exposure to broad-spectrum antibiotics, all of which exacerbate the immunosuppressive milieu (Figure 2). As such, reversing immunoparalysis is a critical therapeutic goal in sepsis management [88,119,120,121].

### 4.6. Pathogen Immune Evasion Mechanisms

Numerous pathogens have developed strategies to evade inflammasome activation, a key component of host innate defense. These mechanisms enable persistence within the host while suppressing inflammatory responses. *Mycobacterium tuberculosis*, for example, uses its ESAT-6 protein to inhibit mitochondrial ROS production—an upstream trigger of NLRP3 activation—thereby preventing inflammasome assembly, reducing IL-1β release, and avoiding immune detection [122,123,124,125].

*Listeria monocytogenes* activates NLRP3 through listeriolysin O–induced mitochondrial damage and mtDNA release. However, it finely regulates this activation, creating a controlled inflammatory environment that supports bacterial replication while limiting host clearance [122,123,124,125].

Viral pathogens such as hepatitis B virus adopt a suppressive approach, directly inhibiting IL-1β maturation to dampen inflammasome signaling and promote immune evasion [122,123,124,125].

Through these strategies—suppressing inflammasome components or modulating activation thresholds—pathogens evade immune surveillance, enabling chronic infection and contributing to the persistent inflammation and immune dysfunction seen in severe sepsis [122,123,124,125].

## 5. Emerging Molecular Regulators and Mechanisms

### Plasma Membrane Rupture and the Role of NINJ1

Plasma membrane rupture (PMR) represents the terminal and irreversible step in lytic forms of cell death such as pyroptosis, necrosis, and secondary apoptosis. This event facilitates the massive release of intracellular proinflammatory mediators—classified as DAMPs—including high mobility group box 1 (HMGB1) and LDH, which in turn amplify systemic inflammation [126].

In the context of sepsis, PMR is a critical determinant of progression toward multiorgan failure. Recent studies have identified Ninjurin-1 (NINJ1), a transmembrane protein previously linked to nerve regeneration, as an essential regulator of PMR [127,128]. Unlike GSDMD-mediated pore formation, NINJ1 contributes to the structural disintegration of the plasma membrane, playing an active—not merely permissive—role in the dissolution process during lytic cell death [126].

Mutagenesis studies in mouse models have shown that while NINJ1 deficiency does not impair inflammasome activation or initiation of cell death, it prevents terminal membrane rupture and inhibits DAMP release [127,129]. This finding highlights the mechanistic dissociation between cell death execution and inflammatory signal propagation, positioning PMR as a molecularly regulated step rather than a passive consequence of cellular breakdown. In the absence of NINJ1, cells undergoing pyroptosis display abnormal vesiculation and fail to complete membrane lysis, reflecting the incomplete execution of terminal rupture [126].

Upon inflammasome activation—particularly via the NLRP3 pathway—GSDMD is cleaved by caspase-1 to generate an N-terminal fragment that forms pores in the plasma membrane [127,129]. However, full-scale membrane rupture and maximal DAMP release require the downstream action of NINJ1. This protein oligomerizes through a conserved extracellular domain, destabilizing the membrane and enabling structural collapse. In sepsis, where excessive and uncontrolled inflammation underlies tissue injury and lethality, NINJ1 represents a novel and promising therapeutic target for attenuating inflammatory damage without interfering with the initial pathogen-sensing or cell death machinery [126].

Recent structural studies have elucidated the molecular basis of NINJ1-mediated PMR. Oligomerization of amphipathic α-helices—particularly involving Lys45, Asp53, and Gly93/95—enables NINJ1 to perforate the membrane independently of GSDMD pores. NINJ1 assembles into hand-in-hand oligomers that form filaments or ring-like “cookie-cutter” structures, actively destabilizing the membrane and promoting DAMP release. Its activation is context-dependent and influenced by osmotic stress and cytoskeletal disruption [130,131]. Importantly, NINJ1-targeted interventions—including monoclonal antibodies (e.g., Clone D1), blocking peptides, and small-molecule inhibitors such as glycine and muscimol—have shown efficacy in reducing inflammation and organ injury in experimental models of sepsis. These agents selectively block membrane rupture and DAMP release without preventing the initiation of cell death, thereby preserving host defense mechanisms while attenuating downstream inflammatory amplification [130,131].

## 6. Therapeutic Strategies Targeting Inflammasomes

Current sepsis management relies on timely administration of antimicrobial therapy, source control, hemodynamic stabilization, and supportive care for organ function [132]. Despite these interventions, sepsis remains a leading cause of morbidity and mortality, underscoring the limitations of existing treatment paradigms. The pathophysiology of sepsis reflects not only a dysregulated immune response to infection but also a complex interplay between hyperinflammation and subsequent immunosuppression. As such, immunomodulation is increasingly recognized as a critical strategy to reduce mortality and improve clinical outcomes.

### 6.1. Pharmacological Inhibitors and Experimental Compounds

A key driver of inflammatory damage in sepsis is the non-canonical inflammasome, mediated by caspase-11 in mice and its human homologs, caspase-4 and caspase-5. This pathway is activated directly by intracellular LPS, independent of Toll-like receptor 4 (TLR4), leading to GSDMD cleavage, pyroptosis, and release of DAMPs. Importantly, it can also amplify inflammation by secondarily activating the canonical NLRP3 inflammasome, establishing a feedforward inflammatory loop [133,134,135].

Experimental models have demonstrated that genetic ablation of inflammasome components—such as NLRP3, caspase-1, caspase-11, or GSDMD—confers protection against sepsis-induced mortality by attenuating systemic inflammation, coagulopathy, and multiorgan dysfunction. These findings have prompted the development of pharmacological inhibitors targeting inflammasome pathways. Notable candidates include MCC950, a selective NLRP3 inhibitor; anti–IL-1β monoclonal antibodies; and emerging GSDMD inhibitors, all of which aim to suppress the hyperinflammatory milieu of sepsis [136,137,138,139,140].

In addition to specific inhibitors, several non-selective agents have shown beneficial effects in preclinical models. Compounds such as baicalin, cortistatin, and sulfur dioxide have been reported to inhibit NLRP3 activation, reduce IL-1β and IL-18 levels, and mitigate oxidative stress and cell death in animal models of sepsis [141]. These findings underscore the potential of targeting inflammasome signaling as a means to rebalance the host immune response.

### 6.2. Extracorporeal Therapies and Hemoadsorption

Beyond pharmacological inhibition, extracorporeal therapies have gained attention as adjunctive strategies to modulate systemic inflammation. Techniques such as hemoadsorption using polymer-based cartridges (e.g., CytoSorb^®^), high-volume hemofiltration, and plasmapheresis aim to remove circulating cytokines and DAMPs, thereby reestablishing immune homeostasis. While these modalities have shown promise in improving hemodynamic parameters and reducing inflammatory mediators, consistent evidence of survival benefit remains limited and requires further validation in large-scale clinical trials [142,143,144].

### 6.3. Immune Stratification and Precision Immunotherapy

The clinical heterogeneity of sepsis underscores the need for precision medicine approaches. Immunophenotyping and biomarker-guided stratification are essential for identifying patients who may benefit from targeted immunomodulation. Biomarkers such as procalcitonin, HLA-DR expression on monocytes, interleukin-10 (IL-10) levels, and transcriptomic profiles are increasingly used to guide individualized treatment strategies. These include the use of inflammasome inhibitors, IL-1 antagonists, immune checkpoint modulators, and antioxidants [145,146,147].

In addition to inflammasome-directed therapy, upstream cytokine modulation represents a complementary approach. Tumor necrosis factor-alpha (TNF-α) and interleukin-6 (IL-6) are key mediators of organ dysfunction in sepsis. Preclinical studies have shown that agents such as infliximab (anti–TNF-α monoclonal antibody) and tocilizumab (IL-6 receptor antagonist) can reduce tissue injury and improve survival in models of sepsis-induced lung and kidney injury [148]. These findings support the strategy of cytokine modulation as a complement to inflammasome-targeted therapy.

Inflammasomes function as central molecular hubs integrating microbial signals, mitochondrial dysfunction, regulated cell death, and sterile inflammation. Their dysregulation in sepsis drives the transition from localized infection to systemic inflammatory response syndrome (SIRS), with devastating consequences for organ function [88,119,121]. Nevertheless, critical questions remain regarding the optimal timing, cellular specificity, and context-dependent effects of inflammasome activity across different sepsis phenotypes. A deeper understanding of their crosstalk with other regulated cell death pathways—including apoptosis, necroptosis, and ferroptosis—will be instrumental in guiding the development of safe and effective immunomodulatory therapies for sepsis [88,119,121] (Table 3).

## 7. Innate Inflammation in Neonates

Neonatal sepsis is a dysregulated systemic inflammatory condition that emerges during a critical window of immune system development. Recent evidence suggests that the neonatal immune response is initially hyporesponsive, followed by a paradoxical hyperinflammatory phase that contributes to multiorgan dysfunction and long-term neurological sequelae [149,150,151,152]. This immune imbalance is linked to insufficient activation of pattern recognition receptors—particularly TLRs—resulting in impaired phagocytosis, antigen presentation, and reduced production of cytokines such as interleukin-12 (IL-12) and IFN-γ [151,152].

As a result, neonates exhibit a predominantly Th2-skewed and tolerogenic immune profile, likely an adaptive legacy of fetal-maternal tolerance. However, this profile proves inadequate in mounting effective responses against invasive pathogens [149,150,151,152]. The immaturity of innate immunity in this population creates a window of vulnerability, particularly against encapsulated bacteria.

Murine models of neonatal sepsis using encapsulated Escherichia coli have shown that pentoxifylline (PTX), a phosphodiesterase inhibitor, can modulate inflammation by suppressing TNF and IL-1β production while enhancing IL-10 levels. Mechanistically, PTX increases intracellular cyclic AMP and inhibits NF-κB–dependent transcription of proinflammatory genes. These effects are significantly more pronounced in umbilical cord blood cells compared to adult immune cells, highlighting developmental differences in immunomodulatory responses. In addition, preclinical studies using aluminum-based adjuvants (alum) have demonstrated that prophylactic activation of the NLRP3 inflammasome enhances innate immune responses and improves survival in neonatal sepsis models. This effect occurs independently of B and T lymphocytes, suggesting a mechanism of trained immunity that enhances pathogen resistance in neonates [150,151].

The pathophysiology of neonatal sepsis involves biphasic immune dysregulation, immune evasion by encapsulated pathogens, cytokine overproduction, epithelial barrier disruption, and context-dependent inflammasome activation [149,150,151,152]. Among these mechanisms, the NLRP3 inflammasome stands out as a promising therapeutic target. Although still in the preclinical stage, PTX and alum represent potential immunomodulatory interventions to restore immune equilibrium and improve outcomes in this highly vulnerable population [149,150].

## 8. Discussion

This review underscores the multifaceted role of inflammasomes as central mediators in the pathophysiology of sepsis. Among these, the NLRP3 inflammasome stands out as a key integrator of both exogenous and endogenous danger signals, orchestrating inflammatory responses, oxidative stress, coagulation, and regulated forms of cell death such as pyroptosis [153,154]. Although less extensively studied in sepsis, other sensors such as AIM2 and IFI16—cytosolic and nuclear DNA detectors, respectively—have emerged as relevant contributors to the host inflammatory response, particularly in the context of viral infections [10,86,155].

Inflammasome activation not only initiates cytokine release and pyroptosis, but also contributes to endothelial dysfunction, immune exhaustion, and immunosenescence—thereby establishing a mechanistic link between acute inflammation and long-term immune dysregulation [113].

While molecular and animal studies have illuminated key aspects of inflammasome signaling, it must be noted that the majority of preclinical data are derived from murine models. These models, although invaluable for deciphering cellular mechanisms, often fail to replicate the immunological and clinical heterogeneity of human sepsis. Additionally, data on non-canonical inflammasomes in specific sepsis phenotypes—such as neonatal sepsis, immunocompromised states, or viral infections—remain limited. The absence of longitudinal studies capturing inflammasome dynamics across sepsis stages further constrains our understanding of their temporal roles. Moreover, although therapeutic strategies targeting inflammasomes—such as NLRP3 inhibition with MCC950 or gasdermin D blockade—have shown promise in preclinical settings, conclusive evidence from clinical trials is still lacking [156,157].

This review builds upon prior work highlighting NLRP3 as a master regulator of inflammatory signaling in sepsis [62], while extending current understanding by addressing lesser-explored sensors like AIM2 and IFI16. Importantly, it reinforces the concept that inflammasome dysregulation not only contributes to local tissue injury but also promotes systemic procoagulant states and immunoparalysis—echoing recent findings at the intersection of inflammation and coagulation in sepsis [5,148].

Sepsis may increasingly be conceptualized as a syndrome of immunometabolic dysfunction, wherein inflammasome activation—particularly NLRP3—interacts with comorbid conditions such as diabetes. NLRP3 drives the maturation and release of IL-1β and IL-18, cytokines implicated in insulin resistance and metabolic derangement [63]. In autoimmune disorders, sustained inflammasome activity promotes T-cell polarization and autoantibody production, perpetuating oxidative stress and chronic inflammation [158,159]. This pre-primed inflammatory state may worsen the host response to infection and accelerate progression to multiorgan failure in sepsis [160].

These insights open multiple avenues for future research. It is essential to identify the specific immune cell subsets expressing inflammasome components during various phases of sepsis, and to define how activation patterns vary by pathogen type, comorbidities, and immune status. The development of dynamic biomarkers to monitor inflammasome activity in real time could enable patient stratification and guide personalized immunomodulatory strategies.

From a clinical perspective, recognizing inflammasomes as integrative nodes linking inflammation, mitochondrial dysfunction, and coagulopathy reorients the therapeutic focus toward restoring immunometabolic homeostasis. This conceptual shift moves beyond the traditional framework of unrestrained inflammation and emphasizes selective immune rebalancing rather than global suppression. Inflammasome-targeted therapies hold promise for advancing precision medicine in sepsis—a syndrome that has historically eluded effective immunologic intervention [161].

Despite promising results in preclinical models, most inflammasome-targeted therapies—such as MCC950, anti–IL-1β antibodies, and GSDMD inhibitors—remain in early-stage clinical development and lack validation in diverse patient populations. While murine models have been instrumental in elucidating inflammasome biology, they do not capture the full complexity of human sepsis, which is influenced by age, genetic background, comorbidities, prior immune exposures, and microbiome composition.

Notably, species-specific differences in inflammasome pathways present translational challenges. For example, the non-canonical inflammasome relies on caspase-11 in mice, while in humans it is mediated by caspase-4 and caspase-5. These differences complicate the extrapolation of mechanistic findings and therapeutic responses from animal models to clinical settings.

Furthermore, many experimental studies overlook the biphasic nature of sepsis, where an initial hyperinflammatory phase is followed by a prolonged state of immunosuppression. Effective immunomodulatory strategies must account for this dynamic trajectory, with timing and context-specific interventions.

These limitations underscore the urgent need for longitudinal, patient-centered studies incorporating immunophenotyping and real-time biomarker assessments to evaluate the efficacy and safety of inflammasome-based therapies. Without such data, there is a risk of misapplication, potentially leading to ineffective or even harmful outcomes in septic patients.

## 9. Conclusions

This integrative review concludes that inflammasomes constitute essential components in sepsis pathophysiology, serving as sensing and effector platforms that translate damage and infection signals into dysregulated inflammatory responses. Among them, the NLRP3 inflammasome stands out as a molecular hub that integrates diverse danger signals and orchestrates the inflammatory cascade in sepsis. Far from being passive mediators, inflammasomes actively regulate key processes such as cytokine maturation, pyroptotic cell death, endothelial dysfunction, oxidative stress, immunothrombosis, and subsequent immune exhaustion. The NLRP3 inflammasome, in particular, emerges as a central integrator of metabolic, mitochondrial, ionic, and damage-associated signals, whose dysregulation drives progression from localized infection to systemic inflammation and multiorgan dysfunction.

Alongside NLRP3, other DNA-sensing inflammasomes such as AIM2 and IFI16 expand the host’s capacity to detect cytosolic and nuclear DNA, playing critical roles in sterile inflammation and viral immunity. These sensors link mitochondrial dysfunction and pathogen recognition to maladaptive immune responses, reinforcing the pathophysiological complexity of sepsis.

In response to the questions posed at the outset of this review, we can affirm that inflammasomes not only amplify systemic inflammation but also modulate downstream pathophysiological events that define sepsis severity. These include disruption of endothelial integrity, activation of coagulation pathways, ROS-mediated cellular injury, and transition into immunosuppression. Their activation is profoundly shaped by host factors such as age, sex, comorbidities, and socioeconomic and environmental conditions—positioning inflammasomes as both molecular and contextual determinants of disease trajectory.

Nevertheless, significant gaps remain in the literature. We must deepen our understanding of the sequential and spatial activation of different inflammasomes across various cell types and sepsis stages. Future research should focus on developing specific biomarkers that reflect real-time inflammasome activity, as well as clinical validation of targeted therapies like selective NLRP3 inhibitors, GSDMD blockers, and extracorporeal immunomodulation strategies.

The clinical application of these findings may establish the foundation for precision medicine in sepsis, aimed at intervening in key points of the inflammatory cascade without compromising host immune defenses, thereby reducing the morbidity and mortality of one of the most lethal and complex syndromes in contemporary medical practice.

## Figures and Tables

**Table 1:** Post-translational modifications required for inflammasome activation in sepsis.

Sensor	Key Post-Translational Modifications for Activation
NLRP3	Deubiquitination, phosphorylation of critical serine residues, reversible sumoylation, interaction with TXNIP, NEK7-induced oligomerization, and ASC recruitment.
AIM2	Direct binding to cytosolic double-stranded DNA does not require intrinsic modifications, but ASC phosphorylation has been described as an indirect regulator.
IFI16	Nuclear acetylation promotes cytosolic translocation and interaction with ASC at damaged chromatin domains; ubiquitination inhibits activity during viral infections.

**Table 2:** Key inflammasome sensors and their stimuli in sepsis.

Sensor	Localization	Activators	Key Pathogens	Downstream Effects
NLRP3	Cytosol	ROS, ATP, mtDNA, K^+^ efflux, lysosomal damage	Bacteria, viral RNA	Caspase-1 → IL-1β, IL-18, GSDMD (pyroptosis), coagulation
AIM2	Cytosol	dsDNA (microbial or host)	*Francisella*, *Brucella*, SARS-CoV	Caspase-1 → IL-1β, pyroptosis
IFI16	Nucleus → cytosol	Viral nuclear DNA	KSHV, HSV	Caspase-1 → IL-1β (especially chronic inflammation)
NLRC4	Cytosol	Bacterial secretion system proteins (e.g., *Salmonella* SipB)	*Salmonella*, *Legionella*	Caspase-1 → IL-1β, pyroptosis

ATP: Adenosine Triphosphate; dsDNA: Double-Stranded DNA; GSDMD: Gasdermin D; HSV: Herpes Simplex Virus; IL-1β: Interleukin 1 Beta; IL-18: Interleukin 18; K^+^: Potassium Ion; KSHV: Kaposi’s Sarcoma-Associated Herpesvirus; mtDNA: Mitochondrial DNA; ROS: Reactive Oxygen Species; SARS-CoV: Severe Acute Respiratory Syndrome Coronavirus; SipB: Salmonella Invasion Protein B.

**Table 3:** Therapeutic strategies targeting the inflammasome in sepsis.

Strategy	Mechanism of Action	Current Status	References
Specific pharmacologic inhibitors	Inhibit key inflammasome components (NLRP3, caspase-1, caspase-11, and GSDMD), reducing inflammation, coagulopathy, and multiorgan failure.	Preclinical models show protective effects.	[136,137,138,139,140]
MCC950 (selective NLRP3 inhibitor)	Directly blocks NLRP3 activation, reducing IL-1β and IL-18 release.	Under experimental development.	[136]
Anti-IL-1β antibodies	Neutralize IL-1β to suppress systemic inflammation.	Currently under clinical evaluation.	[137,138]
GSDMD inhibitors	Prevent gasdermin D-mediated pore formation and pyroptosis, thereby reducing DAMP release.	In preclinical development.	[139,140]
Non-selective compounds	Baicalin, cortistatin, and sulfur dioxide inhibit NLRP3 activation and reduce IL-1β, oxidative stress, and cell death in septic models.	Demonstrated efficacy in animal studies.	[141]
Extracorporeal therapies	Hemoadsorption, high-volume hemofiltration, and plasmapheresis remove circulating cytokines and DAMPs, helping restore immune balance.	Mixed results; more evidence required.	[142,143,144]
Precision immunotherapy	Biomarkers such as procalcitonin, HLA-DR expression, IL-10, and transcriptomics guide personalized immunomodulation.	Advanced clinical research ongoing.	[145,146,147]
Cytokine-targeting agents	Infliximab (anti-TNF-α) and tocilizumab (anti-IL-6 receptor) reduce organ damage and improve survival in sepsis models.	Promising preclinical data.	[148]

## Data Availability

No new data were created or analyzed in this study.
